# Household food security, child dietary diversity and coping strategies among rural households. The case of Kole District in northern Uganda

**DOI:** 10.1016/j.dialog.2023.100149

**Published:** 2023-07-28

**Authors:** Samuel Elolu, Alod Agako, Daniel Micheal Okello

**Affiliations:** a Department of Food Science and Postharvest Technology, Faculty of Agriculture and Environment, Gulu University, P.O BOX 166, Gulu, Uganda; b Department of Rural Development and Agribusiness, Faculty of Agriculture and Environment, Gulu University, P.O BOX 166, Gulu, Uganda

**Keywords:** Child dietary diversity, food security, coping, livelihood diversity

## Abstract

Household food security, feeding practices, dietary diversity and coping strategies to household food insecurity are largely interconnected. Using a cross sectional study approach involving 162 mothers and care givers of children 0-36 months of age in Kole district of northern Uganda, this study examined the household food security status, child dietary diversity and household coping strategies. The study revealed that a slight majority of the households (55%) were food secure although with a low level of child dietary diversity experienced (68.1% for children 6-23 months age group and 55.3% for 24-36 months age group). We found that starch-based foods derived from cereals, roots and tubers were the most predominantly used food group in child feeding (82%), with limited consumption of other essential food groups, notably fruits, vegetables, meats and dairy products (18% combined). Additionally, only 57% of children 0 to 6 months old were receiving exclusive breastfeeding, and the introduction of complementary foods is often delayed and not well planned for those above 6 months of age. Results also showed that a wide range of coping strategies are employed however the major ones were, reliance on less preferred food (54.9%), limiting portions of meals (35.2%), reducing number of meals taken in a day (29%), and gathering wild fruits and harvesting immature crops (29.6%). It was observed that household food security is a strong determinant of child dietary diversity, may influence feeding practices and the range of coping strategies applicable to households when they experience food insecurity. Furthermore, nutritional education, household size and livelihood diversity play a significant role in determining household food security status, child dietary diversity and coping with food insecurity within rural households. In conclusion, addressing household food security, and coping strategies can play an important role in improving child feeding practices and dietary diversity in rural communities.

## 1 Introduction

Persistent undernutrition in early childhood is associated with poor development outcomes, poor health and poor achievement during adulthood [1]. The burden of undernutrition is most prevalent in low income nations where children are usually the most affected [2]. Even within these countries, the burden is not uniformly distributed, with the rural areas most hit by the challenge of undernutrition, largely due to the resource constraints experienced. Existing literature indicates that there is a strong linkage between household food security status and nutritional outcomes of household members, including children [3]. However, studies on household food security have focused on the determinants and welfare outcomes, studied majorly in respect to income and economic outcomes [[4], [5], [6]]. There has been limited attention on investigating the linkage with child feeding practices and coping mechanisms, particularly for households in agrarian rural areas such as those in Uganda. Uganda’s child nutrition research indicates that the country’s children suffer from high levels of under nutrition indicated by 29 % stunting, 4% wasting and a significant level of nutrition related deaths, among children [7,8]. Relatedly, an assessment in northern Uganda indicated that about 24% of households were food insecure, and that only 12% of children aged 6 to 23 months had minimum acceptable diets, with only 26% of children fed as per recommended minimum dietary diversity [9]. Similarly, Nabuuma et al. [10] in a qualitative study reported that dietary diversity remains low and challenging to achieve among rural farmers in Uganda, but specific quantification of dietary diversity is not well addressed, even though there is sufficient evidence that a lack of healthy diets is more detrimental in terms of long-term effects, especially to children. It is well understood that child feeding practices vary across communities and are largely influenced by different socioeconomic conditions prevailing within the communities [2]. Moreover, the feeding practices are key determinants of children’s nutritional status and health. For instance, exclusive breast feeding for six months is known to be associated with reduced mortality and morbidity for children under five years [11]. Furthermore, the risk of malnutrition for children under 2 years is well linked to the feeding practices, notably breast feeding and complementary feeding practices by mothers and/or care givers of children within that age bracket [11]. Similarly, Mattson et al. [12] reported that, the nutritional practices and food habits normally translate directly and indirectly into nutritional and health status of individuals. From a broader perspective, household food security status is a known direct consequence of several household characteristics. Further, in developing countries, most of the households’ food needs are met through own production, and thus food utilization or feeding decisions are majorly made by household heads, mothers or caregivers [13]. As such, it is expected to have a strong bearing on the child dietary diversity, overall child feeding practices as well as coping strategies employed by the care givers in different households.

Though many studies on coping strategies exist, a scan through them reveals that most are focused on general household level coping rather than coping in specific regards to child feeding [[14], [15], [16]]. Coping mechanisms employed by households are critical towards understanding vulnerability of people in the face of food insecurity and could have other far-reaching effects on the dietary diversity situation in the household. For instance, Olaimat et al. [17] reported that food-related coping strategies such as eating less meals per day, consuming smaller amounts of food per day and skipping meals by adults were significantly associated with food insecurity. Similarly, Murendo et al. [18] found that household dietary diversity differed based on the food security status and resilience level of household, with direct influence of the distribution of the available foods among household members such as adults and children. Given that appropriate child feeding has long term consequences on child growth, cognitive development and is associated to poor individual health outcomes even at adulthood, it is apparent that bridging the knowledge gap on the influence of household food security and child feeding practices in a local context is necessary. This study therefore contributes empirical knowledge insights on interrelationships between household food security, child feeding and copying strategies.

## 2 Materials and methods

### 2.1 Study area, study population and sampling

This study was conducted in Kole district, located in northern Uganda. The study area is typically an agrarian rural setting, between latitudes 02^o^ 124’N and 2.400^o^ N; and longitudes 32^o^ 48’E and 32.800^o^E in northern Uganda. Contextually, 92% of the households in the district depend on subsistence farming as a main source of livelihood, with 96% of them engaged in crop farming, the major crops being staples such as cassava, maize, and beans [19]. The study targeted households with children 0-36 months of age and specifically, either the mother or the caregiver of the child within the targeted age group were interviewed in the study. A purposive sampling approach was used to select the mothers and caregivers of children under 3 years old. This was done through the village health teams. In total, eight villages with functional village health teams (with complete and updated records) were selected from two parishes and 20 households from the records having children 0 to 36 months old were selected. An additional 10% of the initial sample was added, to cater for possible data attrition. The Cochran formula as described by [20], was used to determine the sample size.However, for the analysis, only 162 complete data sets (from 162 mothers or caregivers) were considered.

### 2.2 Study design and data collection

A cross sectional survey design was used to collect data from 162 households in the study area. Data on household food security status was collected using the HFIAS scale, with questions, consisting of three domains (categories) in the household questionnaire namely, anxiety and uncertainty about household food supply; food quality; and food quantity intake, related to food availability [21]. Data on child feeding and dietary diversity was obtained using the modified WHO guidelines [22], as previously applied using indicators such as; exclusive breastfeeding (EBF) for the first 6 months; time of initiation of complementary feeding; minimum dietary diversity, minimum frequency of feeding and minimum acceptable diet [23]. To assess response to food shocks by each household, the Coping Strategy Index (CSI) was used, as previously applied, including a weighted list of locally available coping strategies included in the household questionnaire [24,25]. Descriptive data on the general characteristics of the study population was included as the first part of the data collection. Trained enumerators were deployed to conduct a pretest and later final data collection. Prior to data collection, the study sought ethical clearance from Gulu University Research Ethics Committee (GUREC). Further permission to conduct the study was also obtained from the district local government officials and lastly, informed consent was obtained from each of the participants before being engaged in the study.

### 2.3 Data analysis

Data was analyzed using SPSS v25 and STATA v14. Analysis involved both descriptive and inferential statistics. Data on household food security status from the HFIAS scale was used to categorize households as either food secure or food insecure. Data on child dietary diversity was used to categorize children as having either high diverse diet or low diverse diets. Descriptive analysis was conducted using frequencies and means. Regression analyses were performed to assess the factors associated with household food security status, coping strategy and child dietary diversity. Since each of these variables were measured differently, an appropriate model was chosen in each case. In assessing factors associated with household food security status, a binary logistic regression model was fitted. This was because the dependent variable, household food security status was binary in nature with a household either falling in the food secure category (1) or the food insecure category (0). On the other hand, assessing the factors associated with household coping strategy, a multivariate least squares regression analysis was performed since the household coping strategy index, was a continuous variable. Finally, in assessing the factors associated with child dietary diversity, a binary logistic regression model was estimated, since, the variables child dietary diversity was binary in nature, with the child being classified either as having high dietary diversity (1) if child’s 24-hour dietary diversity score was above four food groups or as having low dietary diversity (0) if the child’s 24-hour dietary diversity was four or less food groups. The general specification for the binary logistic regression for assessing factors associated with household food security status and child dietary diversity score is specified in Equation (1).

(1) $$\log\left[\frac{p}{1-p}\right]=\alpha +XB^{\prime }+\mu$$

Where: p is the probability that a given household is food secure in the case of assessing factors associated with household food security status, or the probability that a child has a diverse diet, in the case of assessing factors associated with child dietary diversity.$\;\left(1-p\right)$ = the probability of a household being food insecure or a child having low dietary diversity. α is the regression constant. $B^{\prime }$ is a vector parameter associated with each of the explanatory variables included in each of the models. X is a vector of explanatory variables that can potentially influences the dependent variable. For each of the models, there were a separate set of explanatory variables. Table 1 presents a description and measurement of these explanatory variables. *μ*= stochastic error term. Prior to running each model, a pairwise correlation analysis was performed to help select the variables to be included in each model. Only variables that had a correlation coefficient of less than 0.45 were included in the model. After the analysis, in each case, post-estimation goodness of fit test were performed and where there was poor fit, the necessary adjustments were made. The final model showed good fit in both cases.

**Table 1:** Description of variables used for regression analysis

Variable	Description/Measurement
Dependent Variables	
HFIAS Classification	Binary dependent variable classifying households into 1=food secure, 0=food insecure, basing on the HFIAS
Coping Strategy Index	Coping strategy index for the households
Child Dietary Diversity Score	Number of difference food groups fed to child over a 24-hour period

Independent Variables	
Sex of child	Sex of reference child, 1=male, 0=female
Age of child	Age of the reference child in months
Age of care giver	1=above 6-month old, 0=6-month-old or less
Prenatal Nutrition training	Mother of reference child attended prenatal nutrition training (1=yes, 0=no)
Education	Level of education of mother/care giver
Married	Marital status of caregiver
BF_Less1Hr	Reference child was breast fed within one hour after birth (1=yes, 0=no)
Still_BF	Child is still breast feeding (1=yes, 0=no)
HH_size	Number of members in the household
Nipple_feed	Child was fed using a bottle with nipple in the last 24 hours (1=yes, 0=no)
Other_food	Child is given other foods (1=yes, 0=no)
Mashed_food	24-hour frequency of child’s consumption of mashed or pureed food or solid or semi-solid food as a meal or a snack
Solid_food	24-hour frequency of child’s consumption of solid, semi-solid, or soft foods other than liquids
Multi_livelihood	Household has more than one livelihood sources (1=yes, 0=no)

Note: HFIAS was included in the coping strategy and dietary diversity models, while, coping strategy was also included in the dietary diversity model as independent variables.

Since the logit model does not show direct changes in the dependent variable attributed to the changes in the independent variables, the marginal effects were estimated following logit regression in each case. Estimation of the marginal effects after logit followed Eq. (2).

(2) $$\frac{\partial \mathrm{Pr}\left[y_{i}=1|x_{\mathrm{ij}}\right]}{\partial x_{\mathrm{ij}}}=\frac{\exp\left(x^{\prime }\delta \right)}{1+\exp\left(x^{\prime }\delta \right)}$$

In order to assess the factors associated with household coping strategy, a multivariate least squares regression was estimated following equation (3)

(3) $$\mathrm{CS}=\beta_{0}+\beta_{1}X_{1}+\beta_{2}X_{2}+\ldots +\beta_{n}X_{n}+\varepsilon$$

Where: CS is the household coping strategy index. $\beta_{0}$ is the regression constant. $\beta_{1},\beta_{2},\ldots ,\beta_{n}$ are parameters associated with the respective explanatory variables, indicating the impact of each explanatory variable on the dependent variable. *X*_1_, *X*_2_, … *X*_n_= are explanatory variables that can potentially influence coping strategy. Table 1 presents a description of these explanatory variables ε is the stochastic error term.

Prior to estimating the multivariate regression model, a pairwise correlation analysis was performed to select variables that should be included in the model. Only variables with correlation coefficient of less than 0.45 were included in the model. Post-estimation Ramsey regression specification (RESET) test, showed that the least squares specification was appropriates, while, the variance inflation factor (VIF) analysis showed that all the variables had a VIF of less than 2.1 with an average VIF of 1.37 with the highest of 2.08. This implies that the model did not have any challenges of multi-collinearity. The presence of heteroskedasticity was addressed using the robust option of STATA. The standard error presented are thus the robust standard errors.

## 3 Results

### 3.1 Household characteristics

The summary statistics for household characteristics of study participants are presented in appendix Table A1, Table A2. It shows that 94% of the care givers were biological mothers of the children, with more than half (52%) of the caregivers between 20 – 29 years old. Majority (85%) of the caregivers were married, with more than half (64%) of the care givers having attained only primary school level of education. In this study, almost all the mothers attended antenatal clinics during their last pregnancy (98%), and post-natal clinics (97.5%). During antenatal and postnatal visits, majority of the mothers usually received nutrition trainings (Appendix Table A1). Results also show that there were three main sources of livelihood for the households with majority (94%) of the households earning a living from farming, another 39% earned their livelihood from businesses, while only nine percent (9%) were formally employed. About 40% of the households had multiple sources of livelihood.

The mean age of the caregivers was 27.5 years, with the youngest care giver being 13 years old, while, the oldest was 69 years old. The mean age of the children was 12.7 month, with the youngest child being 1 month and the oldest being 36-month-old. By age group, 21% of the children were below six month old, 70% were between 6 and 23 monts, while, only 9% were above 23 month old. The households had on average five members with about three children. The largest household had 13 members with eight children, while the smallest household had two members with only one child. The mean ratio of number of children to household members was 0.5 (Appendix Table A2), implying that children constituted at least half of the households.

### 3.2 Child feeding practices

Table 2 presents summary statistics for child feeding practices investigated. It shows that 93% of children had ever been breast feed. Of these, 73% were first breastfed within the first hour of birth, while, 88% were still being breastfed at the time of the study. Nearly nine in ten of the children were being fed other foods and drinks other than breast milk, with most of them being fed twice daily. Similarly, 80% of the children were being fed on solid foods other than liquids almost twice daily, with only 40% of the children being fed using nipple bottles.

**Table 2:** Child feeding practices.

Variable	Category	Freq. (%)				p-value
		All children (n=162)	Children 6-month-old or less (n=34)	Children above 6 – 23 month-old (n=113)	Above 23 month (n=15)	
Child was ever breast fed	Yes	151 (93.2)	37 (94.1)	105 (92.9)	14 (93.3)	0.971
	No	11 (6.8)	2 (5.9)	8 (7.1)	1 (6.7	
Length of time from birth to first breast feeding	Less than an hour	118 (72.8)	27 (67.6)	86 (76.1)	9 (60.0)	0.313
	More than an hour	44 (27.2)	12 (32.4)	27 (23.9)	6 (40.0)	
Child is still breast feeding	Yes	110 (67.9)	28 (82.4)	78 (69.0)	4 (26.7)	0.001
	No	52 (32.1)	6 (17.6)	35 (31.0)	11 (73.3)	
Child eat other food and drink, other than breast milk	Yes	143 (88.3)	16 (47.1)	112 (99.1)	15 (100.0)	0.000
	No	19 (11.7)	18 (52.9)	1 (0.9)	0 (0.0)	
Number of times in a day child is fed mashed or pureed food or solid or semi solid food daily	0	18 (11.1)	18 (52.9)	0 (0.0)	0 (0.0)	0.000
	1	19 (11.7)	3 (8.8)	14 (12.4)	2 (13.3)	
	2	75 (46.3)	12 (35.3)	54 (47.8)	9 (60)	
	3 or more	50 (30.9)	1 (2.9)	45 (39.8)	4 (26.7)	
Child eats solids foods other than liquids	Yes	129 (79.6)	10 (29.4)	105 (92.9)	14 (93.3)	0.000
	No	33 (20.4)	24 (70.6)	8 (7.1)	1 (6.7)	
Number of times in a day child eats solid, Semi-solid or soft foods other than liquids daily	0	26 (16.0)	21 (61.8)	3 (2.7)	2 (13.3)	0.000
	1	34 (21.0)	6 (17.6)	25 (22.1)	3 (20.0)	
	2	65 (40.1)	7 (20.6)	53 (46.9)	5 (33.3)	
	3 or more	37 (22.8)	0 (0.0)	32 (28.3)	5 (33.3)	
Child uses nipple bottle feeding	Yes	63 (38.9)	9 (26.5)	45 (39.8)	9 (60.0)	0.080
	No	99 (61.1)	25 (73.5)	68 (60.2)	6 (40.0)	

In order to identify evidence of exclusive breastfeeding, we use the Pearson’s chi square to test whether there were differences in child feeding practices for those that are supposed to be breastfed exclusively (less than six months old), and children who are supposed to be on complementary feeding. Results showed that over 82% of the children below six month were still being breast fed as opposed to only 69% of the those between 6 and 23 month and 27% for those above 23 months old. There were also a significant association between the child eating other foods and drinks other than breast milk and child age categories. In general, results in Table 4 depicts a gradual transition in child feeding practices by child age group.

Majority of the children below 6 months old, were still breastfeeding at the time of the study. However, not all of them were being breastfed exclusively as required (Table 2). Specifically, about 47.1% of children less than six month were being fed on other non-breast milk semi solid and/or liquid foods, while, about 29% were being fed solid foods. On the otherhand, 18% of the children supposed to be under exclusive breastfeeding (less than six months old) were not being breast fed at all the time of the study. Generally, of the 28 children less than six month old, and still being breastfed, about 43% were being fed other non-breast milk liquid foods alongside the breast milk. Where as another 21% were being feed solid foods. In essence, the actual proportion of children that were being breastfed exclusively was only 57%, a high number given that these children were not supposed to be given other foods (Table 3).

**Table 3:** Proportion of children Below 6 months being exclusively breastfed.

Type of non-breastmilk food or drink given to the child	Response	Freq. (n=28)	%
Child eats other mashed food or drink other than breast milk within the last 24 hours	Yes	12	42.9
	No	16	57.1
Child eats solids foods other than liquids	Yes	6	21.4
	No	22	78.6
Child eats either mashed food or solids	Yes	12	42.9
	No	16	57.1

### 3.3 Household food security status

Using the household food insecurity access scale, this study assessed the household food security level. Table 4 presents the frequency distribution of HFIAS questionnaire items. Overall, majority of the households ranked the responses as rarely and sometimes for most of the questions. There are variations in food security status across households. The mean HFIAS level was 11.5 and a median of 13 shows borderline food security situation. In general, the HFIAS classification shows that over 54% of the household would be classified as food secure, while 46% would be classified as food insecure.

**Table 4:** Household food insecurity status.

HFIAS Question		Frequency (percentage)			
		Never	Rarely	Sometimes	Often
Did you worry that your household would not have enough food?		10 (6.2)	48 (29.6)	68 (42.0)	36 (22.2)
Were you or any household member not able to eat the kinds of foods you preferred because of a lack of resources?		16 (9.9)	43 (26.5)	86 (53.1)	17 (10.5)
Did you or any household member eat just a few kinds of food day after day because of a lack of resources?		15 (9.3)	40 (24.7)	85 (52.5)	22 (13.6)
Did you or any household member eat food that you did not want to eat because a lack of resources to obtain other types of food?		27 (16.7)	44 (27.2)	77 (47.5)	14 (8.6)
Did you or any household member eat a smaller meal than you felt you needed because there was not enough food?		30 (18.5)	30 (18.5)	77 (47.5)	25 (15.4)
Did you or any other household member eat fewer meals in a day because there was not enough food?		32 (19.8)	36 (22.2)	82 (60.6)	12 (7.4)
Was there ever no food at all in your household because there were no resources to get more?		75 (46.3)	51 (31.5)	33 (20.4)	3 (1.9)
Did you or any household member go to sleep at night hungry because there was not enough food?		84 (51.9)	55 (34.0)	19 (11.7)	4 (2.5)
Did you or any household member go a whole day without eating anything because there was not enough food?		117 (72.2)	33 (20.4)	11 (6.8)	1 (0.6)
	**Mean**	**Median**	**SD**	**Min**	**Max**
HFIAS	11.47	13.0	5.631	0	24
**HFIAS classification**	**Frequency/Proportion**				
Food Insecure	74 (45.7)				
Food Secure	88 (54.3)				

SD = Standard Deviation.

### 3.4 Coping strategies

Using the coping strategy scale, this paper assessed the coping strategies with respect to food access for the households. Table 5 presents the frequency distribution of the different coping strategies adopted by households. Very few households responded to the strategies as occurring all the time. Majority however had responses of never, hardly at all and once in a while with respect to the different coping strategies. However, the main coping strategies often applied by households in times of food insecurity were relying on less preferred foods (54.9%), limiting or reducing portions of foods for each member (35.2%), resorting to gathering wild foods (29.6) as well as reducing number of meals consumed per day (29%). The mean coping strategy index of 55, and a median of 51.5 with a range of 0 – 167 implied that majority of the households had a relatively better food security situation. However, it doesn’t mean they are totally food secure and thus turned to different measures to cop in cases and times of shortage.

**Table 5:** Coping strategies.

Coping Strategy	Frequency (percentage)				
	Never	Hardly at all	Once in a while	Pretty often	All the time
Rely on less preferred and less expensive foods	6 (3.7)	21 (13.0)	39 (24.1)	89 (54.9)	7 (4.3)
Borrow food, or rely on help from a friend or relative	64 (39.5)	51 (31.5)	40 (24.7)	7 (4.3)	0 (0.0)
Purchase food on credit	67 (41.4)	51 (31.5)	31 (19.1)	12 (7.4)	1 (0.6)
Gather wild food, hunt, or harvest immature crops	28 (17.3)	35 (21.6)	49 (30.2)	48 (29.6)	2 (1.2)
Consume seed stock held for next season	63 (38.9)	38 (23.5)	45 (27.8)	14 (8.6)	2 (1.2)
Send household members to eat elsewhere	94 (58.0)	44 (27.2)	23 (14.2)	1 (0.6)	0 (0.0)
Send household members to beg	102 (63.0)	38 (23.5)	21 (13.0)	1 (0.6)	0 (0.0)
Limit portion size at mealtimes	38 (23.5)	21 (13.0)	43 (26.5)	57 (35.2)	3 (1.9)
Restrict consumption of adults in order for small children to eat	74 (45.7)	45 (27.8)	30 (18.5)	11 (6.8)	2 (1.2)
Feed working members of HH at the expense of non-working members	116 (71.6)	31 (19.1)	13 (8.0)	1 (0.6)	1 (0.6)
Reduce number of meals eaten in a day	56 (34.6)	26 (16.0)	27 (16.7)	47 (29.0)	6 (3.7)
Skip entire days without eating	128 (79.0)	24 (14.8)	7 (4.3)	2 (1.2)	1 (0.6)
	**Mean**	**Median**	**SD**	**Min**	**Max**
Coping Strategy Index	54.75	51.50	41.68	0.00	167.00

### 3.5 Child dietary diversity

Of the seven food items considered, the childrens' diets were dominated by grains, roots and tubers, followed by other liquids other than milk for all age categories, while eggs and meat products were least components of the diets (Table 6). Specifically, for children aged 6-23 months old, over 92% and 93% of their diets were dominated by grains, roots and tubers, as well as liquids other than milk respectively. Similarly, 82.3% and 93.3% of the diets of those children above 23 months was dominated by these two food groups. This was followed by dairy products like yourghurt and milk at 32.7% for age group of 6-23 months and 66.7% for those above 23 motnhs old. On the otherhand, meat products and eggs were the least components of the diets for all the age groups. In the case of those less than 6 months old, liquids other than milk (41.2%) was the most dominant food group, followed closely by grains, roots and tubers (32.4%). While the rest of the food groups combined were less than 30% in their diets. In general, the child dietary diversity classifications showed that only 27.2 % of the children were consuming highly diversified diets, while the rest had less diversified food groups. The discrepancies between age groups was such that diversity was lowest for the chilren less than 6 months of age as expected, while over 68% and 53.3% of those 6-23 months and above 23 months old respectively had low dietary diversity. Comparison of the realtionship between child age groups and consumption of different food groups showed that consumption of all food groups except for meat products was significantly associated with child age category.

**Table 6:** Child dietary diversity.

Child Food group	Proportion of children (Freq (%))			p-value
	Children 6-month-old or less (n=34)	Children above 6 – 23 month-old (n=113)	Above 23 month (n=15)	
Other liquids other than milk	14 (41.2)	93 (82.3)	14 (93.3)	0.000
Grains, roots and tubers	11 (32.4)	105 (92.9)	14 (93.3)	0.000
Dairy products (milk, yoghurt, cheese)	6 (17.6)	37 (32.7)	10 (66.7)	0.003
Fresh meat, fish, poultry and liver/organ meats	1 (2.9)	20 (17.7)	4 (26.7)	0.051
Eggs or any food made with eggs	0 (0.0)	19 (16.8)	3 (20.0)	0.032
Vitamin A-rich fruits and vegetables	1 (2.9)	31 (27.4)	7 (46.7)	0.001
Other fruits and vegetables	3 (8.8)	49 (43.4)	4 (26.7)	0.001

**Classification of child dietary diversity**				
Low Diversity	33 (97.1)	77 (68.1)	8 (53.3)	0.001
High Diversity	1 (2.9)	36 (31.9)	7 (46.7)	

### 3.6 Factors associated with household food security status

Table 7 presents results of the binary logistic regressions for factors predicting household food security status. It shows that sex of child (p < 0.001), age of the child (p < 0.05), age of care giver (p<0.001), antenatal nutrition training (p < 0.001), breast feeding within an hour of birth (p < 0.05), household size (p < 0.001), frequency of feeding child with solid foods (p < 0.001) and livelihood diversity (p < 0.001) were significantly associated with household food security status. Specifically, household with male children were 42% less likely to be food secure as opposed to those with female children. Similarly, households with the child above 6-month old were 38% less likely to be food secure than those with the reference child less than six month, as majority of the children below six months relied on breastmilk. Household with care givers aged between 20 – 29 years, 30 – 39 years and above 39 years were 54%, 56% and 68% less likely to be food secure, respectively, than households with caretakers below 20 years of age. Similarly, households with an additional household member were 11% more likely to be food insecure. Household with mothers who received nutritional training while attending antenatal clinic, were 52% more likely to be food secure. Results also showed that in households where the child was breastfed within an hour of birth, there was a 26% higher chance of being food secure. The daily frequency for feeding children other foods was also important in that an increase in the number of times a child is fed solid foods daily was more likely (27%) associated with being food secure. Additionally, households with multiple sources of of livelihoods were 39% more likely to be food secure.

**Table 7:** Factors associated with household food security status.

Explanatory Variables (Dependent =HFIAS Classification	Logit regression		Marginal effects after logit	
	Coefficient (SE)	p-value	Margin/dy/dx (SE)	p-value
Sex_child (male)	−1.811 (0.055)	**0.001**	−0.418 (0.112)	0.000
age_child_cat (above 6 month)	−1.860 (0.728)	**0.011**	−0.387 (0.119)	0.001
Below 20=Base				
age_20to29	−2.470 (0.686)	**0.000**	−0.539 (0.118)	0.000
age_30to39	−2.652 (0.792)	**0.001**	−0.563 (0.117)	0.000
age_above39	−5.531 (1.324)	**0.000**	−0.678 (0.057)	0.000
Nutr_training	2.395 (0.608)	**0.000**	0.517 (0.095)	0.000
BF_Less1Hr	1.077 (0.463)	**0.020**	0.263 (0.108)	0.015
HH_size	−0.444 (0.145)	**0.002**	−0.109 (0.036)	0.002
Nipple_feed	0.678 (0.473)	0.152	0.163 (0.111)	0.141
Mashed_food	−0.499 (0.323)	0.122	−0.122 (0.079)	0.122
Solid_food	1.112 (0.352)	**0.002**	0.273 (0.087)	0.002
Multi_livelihood	1.749 (0.554)	**0.002**	0.393 (0.106)	0.000
Constant	2.908 (1.178)	**0.014**		
Number of observations	162			
Wald chi^2^(12)	46.87	0.000		
Pseudo R^2^	0.4207			
Log likelihood	−64.704			
Goodness of fit Pearson chi2(148)	141.24	0.4207		

SE = Standard Error (in parentheses); Variable descriptions follows Table 1. Values in bold indicate statistical significance at p < 0.05.

### 3.7 Factors associated with household coping strategy

Table 8 presents regression results for predictors of coping strategy index (CSI). Results show that household size had a positive significant effect on CSI, while, nipple feeding, livelihood diversity and HFIAS had a negative significant effect on CSI. Child caregivers’ age, level of education, marital status, nutritional training, feeding child with other foods other than milk and length of time to first breastfeeding did not have any significant influence on CSI. Households with an additional member were likely to have their CSI higher by 4.4 units (p < 0.05). Households with children fed using nipple bottles had a 15 units lower CSI than those who do not (p < 0.05), while, households with more than one livelihood source also have a 15-unit lower CSI than those with only one livelihood source (p < 0.05). As expected, food secure households had a 30-unit lower CSI as compared to the food insecure households (p < 0.001).

**Table 8:** Factors Associated with coping strategy index.

Variable	Coefficient (SE)	p-value
Age_caregiver	−0.170 (0.374)	0.649
Post_pri	1.861 (6.103)	0.761
Married	−9.579 (7.968)	0.231
Nutr_training	1.293 (7.658)	0.866
Other_food	9.530 (8.786)	0.280
BF_Less1Hr	−3.004 (6.177)	0.627
HH_size	4.429 (1.958)	0.025
Nipple_feed	−14.883 (5.850)	0.012
Multi_livelihood	−15.277 (5.978)	0.012
HFIAS_CAT	−30.286 (7.090)	0.000
Constant	63.838 (17.820)	0.000
Number of observations	162	
F (10, 151)	11.84	
Prob > F	0.000	
R-squared	0.396	
Ramsey RESET test	F (3, 148)	
	1.86	
Mean VIF = 1.37, Max VIF 2.08		

SE = Standard Error (in parentheses); VIF=Variance inflation factor; RESET = Regresion specific test; Variable descriptions follows Table 1.

### 3.8 Factors associated with child dietary diversity

The binary logistic results for predictors of child dietary diversity are presented in Table 9. Results show that the child age, age of care giver, prenatal nutrition training, household size, household food security status had a positive and significant effect on child dietary diversity, while, the number of children in the household and coping strategy had a negative and significant effect on child dietary diversity. On the other hand, marital status, sex of child, child breast feeding status, nipple feeding and livelihood had no significant influence on child dietary diversity. Specifically, an increase in the age of the child is expected to increase the dietary diversity by 2% (p < 0.001). Children with care givers between the age of 30 – 39 had a 11% higher dietary diversity than those with care givers aged below 20 years (p < 0.05). The other age categories did not differ significantly in child dietary diversity. Similarly, where mothers had received nutritional training during prenatal visits, the child would have a 10% higher dietary diversity (p < 0.05). An increase in household size would increase child dietary diversity by 7% (0.05) while food secure households had a 20% higher child dietary diversity than food insecure households (p < 0.01). With more children, a household would be associated with 3% lower child dietary diversity (p < 0.05). Similarly, households with higher CSI was associated with 0.3% lower child dietary diversity (p < 0.001).

**Table 9:** Factors associated with child dietary diversity.

Variable	Logistic regression		Marginal effects after logit	
	Coefficient (SE)	p-value	Margin/dy/dx (SE)	p-value
Age_of_child	0.171 (0.045)	**0.000**	0.016 (0.005)	0.000
Age of Caregiver (below 20 = base)				
age_20to29	1.129 (0.789)	0.152	0.108 (0.072)	0.136
age_30to39	1.967 (0.989)	**0.047**	0.280 (0.174)	0.106
age_above39	0.921 (1.432)	0.520	0.117 (0.228)	0.607
Married	1.202 (0.928)	0.195	0.084 (0.045)	0.065
Sex_child	0.609 (0.502)	0.224	0.059 (0.052)	0.262
Nutr_training	1.469 (0.750)	**0.050**	0.102 (0.042)	0.015
Child_number	−0.739 (0.337)	**0.028**	−0.071(0.030)	0.017
HH_size	0.680 (0.281)	**0.016**	0.065 (0.023)	0.004
Still_BF	0.907 (0.809)	0.262	0.077 (0.062)	0.209
Nipple_feed	0.622 (0.488)	0.203	0.063 (0.054)	0.244
Multi_livelihood	−0.100 (0.548)	0.855	−0.009 (0.052)	0.854
HFIAS_CAT	2.064 (0.730)	**0.005**	0.197 (0.088)	0.025
Coping strategy index	−0.030 (0.011)	**0.006**	−0.003 (0.001)	0.002
Constant	−9.743 (2.770)	0.000		
Number of observations	162			
Wald chi^2^(12)	51.06	0.000		
Pseudo R^2^	0.418			
Log likelihood	−55.192			
Goodness of Fit Pearson chi^2^(148)	121.81	0.936		

SE = Standard Error (in parentheses). Variable descriptions follows Table 1.Values in bold indicate statistical significance at p < 0.05.

## 4 Discussions

We examined the factors associated with household food security status, child dietary diversity and household food insecurity coping strategies in a typical rural agrarian community. Considering mothers and care givers from househols having children aged between one and thirty-six months old, the study revealed that children in that category made up at least half of the household size, with about three quarters of the children above 6-months old. Whereas exclusive breast feeding is essentially advised for children from birth to 6-month-, results from this study points that nearly half (43%) of the children under 6 months of age did not receive exclusive breast feeding. Children in this age category were additionally fed on mashed and or other liquid foods, coming from other food groups such as other liquids other than milk, grains, roots and tubers and minimally other dairy products. These results reflect similarly to the other recent studies on exclusive breast feeding that found 43.9% and 42.8% prevalence of exclusive breast feeding among different categories of mothers of children under 6 months of age in northern and central Uganda respectively[26,27]. On the other hand, although complementary feeding is safe to introduce after six months, this study reveals that the types of foods fed to children above 6 months in this case was predominantly from high starch sources such as grains, roots and tuber-based foods. It is well documented that the household feeding decisions and practices can greatly influence the nutritional outcomes of children and infants. Specificially, previous reports have emphasized that appropriate feeding practices are critical towards achieving positive child health and nutrition outcomes [11]. Therefore, although the current study did not investigate the nutritional status of children in the community, the observations made on non-exclusive breast feeding and low dietary diversity could explain the high prevalence of child malnutrition as reported in the region [28].

It is apparent that the ability to provide good nutritional care would be a consequence of the overall household food security status. As demonstrated in this study, the household food security status was a strong determinant of the feeding and child dietary diversity observed. The household food security status as measured in this study is largely based on the accessibility dimension [29], indicating that households with limited access to food would have lower levels of diversity. In fact, most of the highly nutritious food groups that are rather expensive and less affordable in the rural contexts were limitedly consumed among the households. As such, children consumed less of such food items like meat, fish and eggs. Consequently, although it was not the subject of this study, it could be expected that the result of this observations is poor nutritional outcomes and deficiencies associated to less consumption of foods such as eggs, meat, poultry, and vitamin A rich fruits and vegetables.

Appropriate initiation and continuation of breast feeding at early childhood is critical for the good nutritional wellbeing of the child [30]. In this study we observed that majority of children obtained breast milk within one hour after birth and were maintained on breast milk for up to between 12 to 23 months, however, the timing of introducing other foods other than milk was rather unclear. We could not obtain this information with certainity from the caregivers, as it would require a longer recall period of reference. Additionally, there was no strong consistency on the type of non-milk foods given to children. For a typical agrarian rural community like the one in this study, the foods were largely starch based. Moreover, reports indicate that complementary foods are often introduced too early or too late [31]. In the case of this study, even when they are introduced at the right age, they are most often nutritionally inadequate, with many of the available and accessible foods inherently poor in protein and micornutritent content. Although exclusive breast feeding is recommended for infants up to 6 months old, it should be noted that milk consumption beyond 6 months is still essential. For instance, milk is a rich source of protein required for growth and development and secondly, younger children are yet not having fully developed digestive systems to completely rely on other solid food alternatives [32]. In this study, we found that nearly half of the children below 6 months were actually not exclusively breast fed, implying that they were exposed to other foods earlier than 6 months as recommended in infant feeding. This could be associated to many factors, including inadequate breast milk due to physiological status of mothers[33], or in some cases where the care giver is not the biological mother of the child. Further, we found that about 31% of the children beyond 6 months were not receiving breast milk anymore. Additionaly, consumption of dairy products was also just at about 33% for children between 6 and 23 month. Although, consumption of dairy products relatively increased among children older than 23 month. Essentislly, low consumption of dairy products implies that the children are at risk of missing the nutritional benefits of milk aforementioned. This is especially of concern given that the complementary foods available are mainly of the type known to be deficient in proteins and essential micronutrients for children in the age category.

It is well emphasized that children should be fed both on balanced and nutritionally diverse diets [34,35]. This is especially important for children above 6 months of age who are expected to be introduced to complementary foods and other meals. However, our study reveals that majority of the children above 6 months old were receiving a diet of low diversity. This could be attributed to the fact that the households here are predominantly peasants and largely obtain their food from their own production. Moreover, the main staples in the region in which the study was conducted are grains (maize), and root crops (cassava). This is an observation previously raised in related studies examining food access and child feeding dynamics in rural agrarian settings [36,37]. The essential implication of this observation is that integrating nutrition in the household production planning would be crucial towards enhancing child nutrition as well as dietary diversity. Though it was observed that about half of the households were food secure, the critical factors associated with household food security status were the age of care takers, nutritional training, household size and livelihood diversity. By way of inference, older care givers were less capable of providing diverse diets largely due to limited livelihood options, while larger households presented a greater burden towards the food needs of the households, hence affecting child feeding. Relatedly, previous studies suggested that more youthful mothers or child caregivers may participate in broader economic activities such as small trade which can widen their access to more and better diverse foods [38]. On the otherhand, as previous scholars such as [[39], [40], [41], [42]] emphasized the need for nutritional education, we observe in this study that integration of nutritional training in antenatal care programs greatly influences the feeding practices, dietary diversity and has a strong positive association with household food security. It is therefore apparent that nutritional education should be an integral part of training for mothers and caregivers of infants and young children.

Generally, we observe that household food security is a strong determinant of child dietary diversity and may influence feeding practices and the range of coping strategies applicable to households when they experience food insecurity. Essentially, the major of household coping strategies were; reliance on less preferred foods, limiting portions of meals, reducing number of meals taken in a day, gathering wild fruits, and harvesting immature crops. These strategies may have specific effect on the outcome of food insecurity towards individual groups in the household namely children, adults, women, and elders among others. For instance, elders and adults may be deprived of adequate food as children may end up being prioritized with the limited food available. Similarly, pregnant women may end up receiving less food than recommended due to inadequacies both in terms of reduced portions available and shifting priorities. These observations were also previously reported in some cases where effects of food security on household food distribution was studied [5]. The aforementioned strategies further lend credence to the role of other food sources and their potential contribution to household food and nutrition. Such sources include wild foods, particularly fruits and vegetables that are still quite available in rural communities such as the study area in northern Uganda [43]. It is worth noting that most of the coping strategies are rather negative actions that are likely to result into further restricted diets and hasten the problem of low dietary diversity. This observation corroborates the findings of Olaimat et al. [17], who found that food coping strategies directly influence dietary diversity in households. Although these strategies may vary from one community to another, it is apparent that there is no specific strategy employed by child caregivers only to target maintaining good child feeding. By inference, this implies that initiatives to improve child feeding practices and dietary diversity should not only focus on improving the overall household food and nutrition security status, but include other components such as provision of nutritional education, and improving or widening household level livelihoods options. One limitation in our study is that we did not collect data over different seasons of the year. In fact, our data was collected in the month of June, typically when the first early food harvests of the year start coming up in the study area. Considering seasonality could be important in terms of household food security, dietary diversity and coping dynamics particularly in such agaragiran settings. Therefore, studies across different seasons should be explored in future.

## 5 Conclusion

Representing a typical rural agrarian community, this study indicated a broad level relationship between household food insecurity, dietary diversity and associated coping strategies. It is evident that most of the diets in this area are largely staple based, mainly dominated by grains, roots and tubers, with evidence showing limited diversity of diets accessible for children. We found that variations in status of household food security is significantly associated with infant or child dietary diversity. Additionally, it is clear that nutritional training, household size and livelihood diversity are critical factors associated with household food security status and thus diversifying the livelihoods options, provision of nutritional education as an integral part of livelihoods and social development initiatives would certainly contribute to better household food security and more diverse diets.

At infant and early childhood stages, it is apparent that exclusive breast feeding is intermittently practiced in such rural areas. Similarly, for most households, the introduction of complementary foods is often delayed by many mothers and child care givers. Finally, in terms of coping strategies, although reliance on less preferred food, limiting portions of meals and reducing number of meals taken in a day were the predominant coping mechanisms among the caregivers, it is apparent that there is no single strategy that can be sufficient in isolation. Therefore, building broad based resilience of households to food insecurity is the most desirable approach to ensure diverse healthy diets and preserve food security.

## Funding

This research received no external funding

## CRediT authorship contribution statement

**Samuel Elolu:** Conceptualization, Methodology, Formal analysis, Investigation, Data curation, Writing – original draft, Writing – review & editing. **Alod Agako:** Conceptualization, Methodology, Formal analysis, Investigation, Data curation, Writing – review & editing. **Daniel Micheal Okello:** Conceptualization, Methodology, Formal analysis, Investigation, Data curation, Writing – review & editing.

## Declaration of Competing Interest

The authors declare no conflict of interest.

## Figures and Tables

**Table A1:** Descriptive statistics for household characteristics.

Variable	Category	Frequency	Percent (N=162)
Care giver is mother of child	Yes	152	93.8
	No	10	6.2
Age of Caregiver	Below 20 years	24	14.81
	20 – 29 years	85	52.47
	30 – 39 years	38	23.46
	Above 39 years	15	9.26
Marital status of care giver	Single	9	5.6
	Married	138	85.2
	Divorced	15	9.3
Education level of care giver	None	15	9.3
	Primary	103	63.6
	O' level	31	19.1
	A' level	3	1.9
	Tertiary	10	6.2
Mother attended antenatal clinics	Yes	159	98.1
	No	3	1.9
Mother received nutrition trainings during Antenatal visits	Yes	130	81.8
	No	29	18.2
Mother attends post-natal clinics	Yes	158	97.5
	No	4	2.5
Mother receives nutrition and feeding practices training during post-natal visits	Yes	125	79.1
	No	33	20.9
Farming is source of livelihood	Yes	152	93.8
	No	10	6.2
Business is source of livelihood	Yes	63	38.9
	No	99	61.1
Employment is source of livelihood	Yes	16	9.9
	No	146	90.1
Livelihood diversity	Yes	63	38.9
	No	99	61.1

**Table A2:** Age and household size.

Variable	Mean	SD	Min	Max
Age of the care giver (years)	27.52	9.47	13	69
Age of the child (month)	12.71	7.24	1	36
Household size	5.46	2.26	2	13
Number of children in the household	2.87	1.89	1	8
Ratio of Number of children to household size	0.50	0.17	0.10	0.89

## Data Availability

Data can be availed upon request to the corresponding author.
